# Positive Selection within the Schizophrenia-Associated GABA_A_ Receptor β_2_ Gene

**DOI:** 10.1371/journal.pone.0000462

**Published:** 2007-05-23

**Authors:** Wing-Sze Lo, Zhiwen Xu, Zhiliang Yu, Frank W. Pun, Siu-Kin Ng, Jianhuan Chen, Ka-Lok Tong, Cunyou Zhao, Xiaojing Xu, Shui-Ying Tsang, Mutsuo Harano, Gerald Stöber, Vishwajit L. Nimgaonkar, Hong Xue

**Affiliations:** 1 Department of Biochemistry, Applied Genomics Laboratory and HKH Bioinformatics Center, Hong Kong University of Science and Technology, Clear Water Bay, Hong Kong, China; 2 Graduate program of Atmospheric, Marine, and Coastal Environment, Hong Kong University of Science and Technology, Clear Water Bay, Hong Kong, China; 3 Graduate Program of Bioengineering, Hong Kong University of Science and Technology, Clear Water Bay, Hong Kong, China; 4 Department of Neuropsychiatry, Kurume University School of Medicine, Fukuka, Japan; 5 Department of Psychiatry and Psychotherapy, University of Würzburg, Würzburg, Germany; 6 Departments of Psychiatry and Human Genetics, University of Pittsburgh School of Medicine, and Graduate School of Public Health, Pittsburgh, Pennsylvania, United States of America; The Wellcome Trust Sanger Institute, United Kingdom

## Abstract

The gamma-aminobutyric acid type-A (GABA_A_) receptor plays a major role in inhibitory neurotransmissions. Intronic SNPs and haplotypes in *GABRB2*, the gene for GABA_A_ receptor β_2_ subunit, are associated with schizophrenia and correlated with the expression of two alternatively spliced β_2_ isoforms. In the present study, using chimpanzee as an ancestral reference, high frequencies were observed for the derived (D) alleles of the four SNPs rs6556547, rs187269, rs1816071 and rs1816072 in *GABRB2*, suggesting the occurrence of positive selection for these derived alleles. Coalescence-based simulation showed that the population frequency spectra and the frequencies of H56, the haplotype having all four D alleles, significantly deviated from neutral-evolution expectation in various demographic models. Haplotypes containing the derived allele of rs1816072 displayed significantly less diversity compared to haplotypes containing its ancestral allele, further supporting positive selection. The variations in DD-genotype frequencies in five human populations provided a snapshot of the evolutionary history, which suggested that the positive selections of the D alleles are recent and likely ongoing. The divergence between the DD-genotype profiles of schizophrenic and control samples pointed to the schizophrenia-relevance of positive selections, with the schizophrenic samples showing weakened selections compared to the controls. These DD-genotypes were previously found to increase the expression of β_2_, especially its long isoform. Electrophysiological analysis showed that this long β_2_ isoform favored by the positive selections is more sensitive than the short isoform to the inhibition of GABA_A_ receptor function by energy depletion. These findings represent the first demonstration of positive selection in a schizophrenia-associated gene.

## Introduction

Schizophrenia is one of the most debilitating mental disorders, afflicting peoples of all countries and cultures with about 1% lifetime risk [1]. Schizophrenics suffer perturbations in thought processes that manifest as hallucinations, delusions, disordered thinking, unusual speech or behavior, and social withdrawal. Schizophrenia tends to run in families with an estimated inheritability of 60–85% [2]. A feature of the disease is a prominent impairment in cognitive functions, especially language-related functions that are unique to mankind. Understanding the etiology of schizophrenia therefore not only paves the way to effective therapeutic treatment of the disease, but may also provide important insight into the nature of human cognition.

Over the past several years, advances in genomics have made possible an initial delineation of the genetic mechanisms of schizophrenia. DNA sequence polymorphisms in a number of genes are found to be associated with the disease [3]. One of the strongest associations is that discovered by our laboratory in the single nucleotide polymorphisms (SNPs) and haplotypes in introns 8 and 9 of *GABRB2* [4]. *GABRB2* codes for the β_2_ subunit of GABA_A_ receptor, and is part of a cluster of genes on chromosome 5q34 for the GABA_A_ receptor, the major inhibitory neurotransmitter-gated channel receptor family in the central nervous system (CNS) [5]. Our initial findings from *Han* Chinese have since been validated by additional samples from the Chinese [6], Portuguese [7], German [7]–[9] and Japanese [9] populations, although conflicting results from Japanese [10] and German [11] populations have also been reported.

As in the case of most other genes underlying complex disorders, the schizophrenia-associated DNA sequence polymorphisms in *GABRB2* are located in the non-coding regions, and therefore not immediately evident in their functional implications. However, recent studies in our laboratory demonstrated that the schizophrenia-associated DNA sequence polymorphisms in *GABRB2* are correlated with under-expression in schizophrenic brains of two alternatively spliced forms of mRNA coding for two previously reported isoforms of β_2_ subunit that differ in length by 38 amino acid residues including a potential Ser/Thr phosphorylation site [12]. The longer isoform was also found to be more prone than the shorter isoform toward current run-down induced by repetitive activation. This finding of a genotype-dependent electrophysiological alteration, establishing an explicit relationship between genotype and potential neuronal behavior, represents the first instance of such relationships identified among the schizophrenia-associated genes.

## Results

### A human-specific Alu insertion

**Figure 1:**
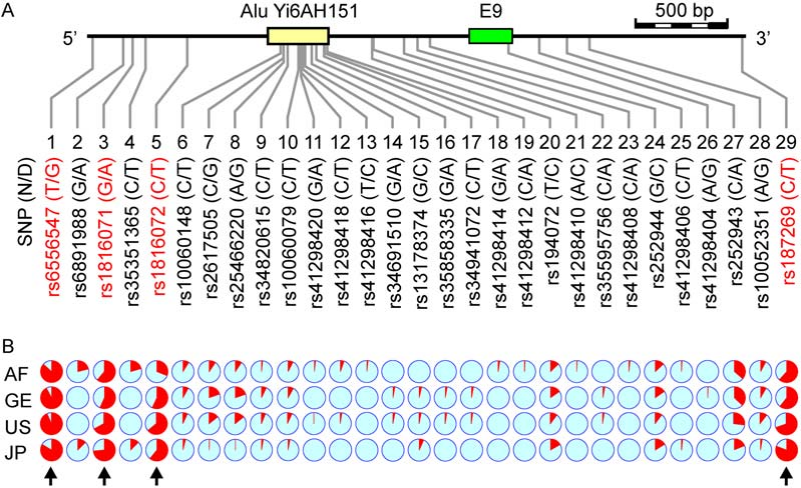
SNP positions and allele frequencies. **(A)** Positions of all twenty-nine SNPs in a 3551-bp region of schizophrenia-associated *GABRB2* from base 160,689,203 to base 160,692,753 of chromosome 5 contig NT_023133.12 are shown to scale. Solid horizontal lines represent introns flanking Exon 9 (green box) and the human specific Alu insert Yi6AH151 (yellow box), which spans bases 160,691,468 and 160,691,782. The ID numbers of SNPs with prefixes “rs” or “ss” are labeled according to the Single Nucleotide Polymorphism database (dbSNP; www.ncbi.nih.gov/SNP). The two alleles for each SNP are shown in parentheses following the SNP ID number in the format of (ancestral base)/(derived base). **(B)** Population allele frequencies of each SNP are represented by pie-charts for the AF, GE, US and JP populations, where the cyanine wedge indicates the frequency of the ancestral allele (N), and the red wedge the frequency of the derived allele (D). The four SNPs with high derived allele frequencies (high-D SNPs) are indicated by arrows and red ID Number.

Based on available genomic sequences of humans and a number of primates, an evolutionary analysis was conducted in the present study comparing human and non-human primate sequences in the schizophrenia-associated *GABRB2* region in order to detect any sequence signatures of human-lineage specific changes that could be indicative of natural selection and therefore functional significance. The non-human primate sequences homologous to the 3,551-bp genomic region of human *GABRB2* were either experimentally obtained or retrieved from the database (see Methods) (Figure 1A). The comparison revealed a human specific 314-bp insertion within this otherwise highly conserved non-coding region of *GABRB2* (Figure S1).

By sequence homology this human-specific insert belongs to the Yi6 sub-group of Alu (Figure S2), a primate-specific family of short interspersed mobile elements with over one million members in the human genome [13]. This insert, which is not identical to any known Alu sequence, is named Yi6AH151 in accordance to Batzer's Alu nomenclature, thereby adding to the 150 known Alu Yi6 elements [14]. It is absent from the non-human primates, but present in all the human subjects analyzed. Therefore it likely represents an insertion into the human lineage prior to human population subdivisions, rather than a deletion from the chimpanzee lineage. That the two SNPs rs34820615 and rs10060148 in Yi6AH151 were found to be associated with schizophrenia [9] suggests the involvement of this Alu in schizophrenia etiology. Possible Alu involvements also have been reported for other diseases [15].

### High sequence diversity in a conserved non-coding region

Re-sequencing was performed on the 3,551-bp genomic segment in the neighborhood of Yi6AH151 for a total of 633 unrelated non-schizophrenic individuals of the four ethnically diverse human populations African (AF), German Caucasian (GE), USA Caucasian (US), and Japanese (JP). A total of 29 SNPs were identified, 15 in the 314-bp Alu Yi6AH151 and 14 on its flanking sequences. Using the chimpanzee and the Yi6 subfamily consensus sequences as references, the two alleles of each of the 29 SNPs could be determined as being the ancestral (N) or derived (D) allele (Figure 1A and Table S1). Based on the N or D status defined, four of the twenty-nine SNPs were found to be exceptionally high in D-allele frequency (>50%), and designated as high-D SNPs (Figure 1B and Table S1). Their high D-allele frequencies signaled the occurrence of positive selection. The four high-D SNPs are in descending order of D-allele frequencies rs6556547, rs187269, rs1816071 and rs1816072. Since rs1816072 exhibits the lowest D-allele frequency among them, it could represent the youngest of the four.

The evolutionary divergences between species, and the diversities within the different human populations were scrutinized for any departure from neutrality. The 0.82% SNP density in the region was found to far exceed the genome-wide and locus-specific densities for the human genome (0.11–0.08%) [16]. Since this region falls within one of the recombination hotspots identified in the HapMap project (Figure S3), the high SNP density observed might have originated from high recombination activities.

In contrast, the 0.74% sequence mismatch between human and chimpanzee in the region is lower than the human genome average of 1.23% nucleotide divergence [17]. This cross-species conservation was suggestive of purifying selection, which is not unexpected for an exon-proximal genic region. This combination of low human-chimpanzee divergence and high intra-human diversity departs from the expectations of neutral evolution (*P* = 0.0013 in the Hudson, Kreitman and Aguadé's test [18]), and points to the occurrence of human-specific events in this otherwise evolutionarily conserved non-coding region [19], [20], resulting in accelerated evolution within the human lineage.

### Ancestral and derived haplotype groups

Frequency spectrum summary statistics were employed to test for any departure from neutral evolution in the region. Based on either the datasets pooled from all four populations, or the datasets of individual populations, positive selection was indicated by some demographic models in the Fay and Wu's *H* test, which takes the ancestral status of polymorphism sites into consideration and is sensitive to an excess of derived alleles. However, neither Tajima's *D* nor Fu and Li's *D* and *F* tests detected significant positive selection in the region (Table S2).

**Figure 2:**
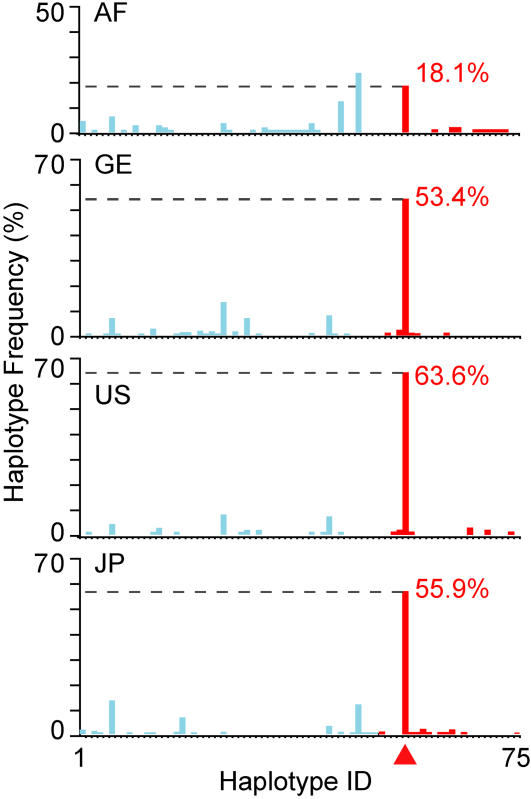
Haplotype frequency distribution in the four populations (refer to Figure 1 legend for abbreviations). Each haplotype is assigned into either the ancestral (HG-N; cyanine) or derived (HG-D; red) group according to its allelic status at rs1816072, likely the youngest high-D SNP. Details of the compositions and frequencies of different haplotypes are shown in Table S3.

**Figure 3:**
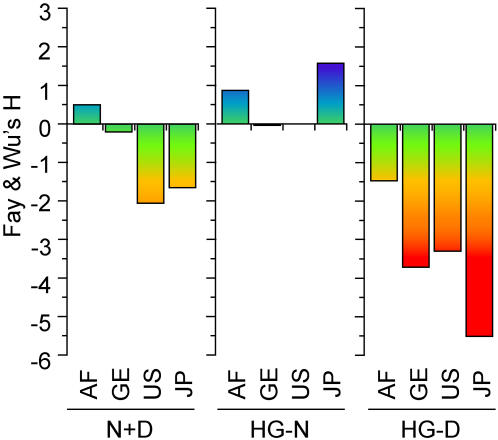
Fay and Wu's *H* values for both HG-N and HG-D haplotypes are plotted for AF, GE, US and JP in thermal scale. Similar plots for Tajima's D and Fu and Li's D are presented in Figure S4.

Accordingly, further investigations of potential positive selection were conducted on separate haplotype groups in accordance to Evans *et al* [21]. Using the allelic state of rs1816072, the youngest of the four high-D SNPs, as defining criterion, all the haplotypes containing its N allele were assigned to haplotype group-N (HG-N; 42.9%), and all those containing its D allele were assigned to haplotype group-D (HG-D; 57.1%) (Figure 2 and Table S3). These HG-N and HG-D haplotype groups were sufficiently similar in size to allow statistically acceptable comparisons. HG-N and HG-D were vastly dissimilar with respect to sequence diversity. The haplotype (*H*_d_) and nucleotide (π) diversities of HG-D were much lower than those of HG-N (Table S2). HG-N displayed a highly diversified membership of fifty different haplotypes (H1 to H50), all of relatively low frequencies (Figure 2 and Table S3). H6, the most abundant member of this group, amounted to a frequency of only 8.5%. In contrast, HG-D contained only twenty-five member haplotypes (H51–H75). H56, its highest-frequency member comprising the D alleles of the four high-D SNPs together with the ancestral alleles of the other 25 SNP sites (Table S3), was present in 51.7% of total N+D copies, or 90.5% of the D copies. This extraordinary predominance by a single haplotype confirmed to the occurrence of exceptionally strong positive selection for H56 and its four constituent D alleles. The differences between HG-N and HG-D in the Fay and Wu's *H* value populations were non-random for all three non-AF populations (Table S2: GE and JP *P*<0.00001; US *P* = 0.07). The mutational spectra in the genealogies of these two classes of haplotypes are therefore sharply different (Figure 3 and Figure S4).

### Demographic evidence against neutral evolution

To distinguish further whether the very high H56 frequency is the outcome of positive selection or demographic effects, coalescence-based simulations were performed. The null hypothesis that a haplotype carrying four or more D alleles could achieve a frequency equal to or higher than the observed H56 frequency in each of the AF, GE, US and JP populations under neutral evolution was tested (Table S4). For this purpose, thirty-three different demographic models embodying population growth [21], population bottlenecks [21], [22], population substructure [21], population growth plus substructure [23], and structured ancestral population with subsequent bottlenecks and expansion [24], were employed. For the AF samples, this null hypothesis was rejected with the Garrigan and Hammers' low-migration model (*P* = 0.00380–0.00420) and Voight's models with most severe bottleneck (*P* = 0.00118–0.00052). As well, it was marginally rejected with the ancient population expansion model (*P* = 0.06153), the extended growth core model with mild bottleneck (*P* = 0.06780) and Voight's bottleneck with modest bottleneck (*P* = 0.06143), although it could not be rejected with the other models. For all three non-AF populations the null hypothesis was rejected for all of the demographic models analyzed except for JP in the case of Model 10 incorporating the most severe bottleneck. These results provided compelling evidence for positive selection acting on H56.

**Figure 4:**
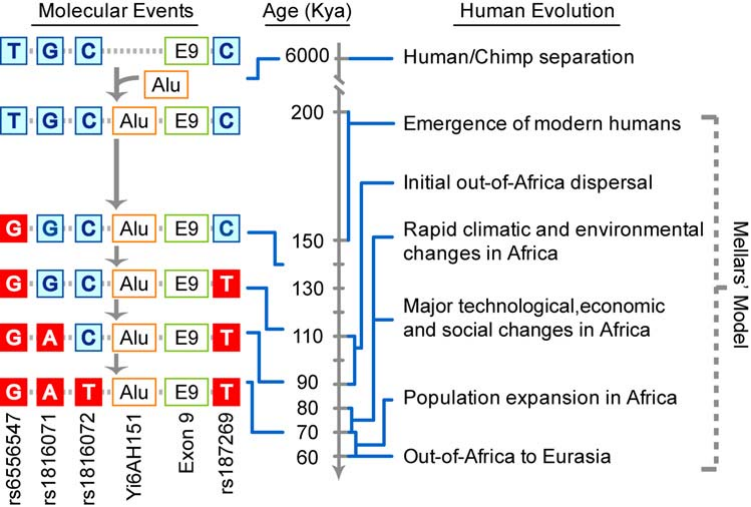
Proposed molecular evolution events in relation to time frame of modern human origins and dispersal from Africa. The age estimates of the four high-D SNPs are shown on the left of this figure. Relevant human evolution milestones, including divergence from chimpanzee and modern human evolution events delineated in Mellars' model [29], are represented on the right. Yi6AH151 is represented as Alu.

While any positive selection acting on the region surrounding Yi6AH151 would preclude a reliable dating of the SNPs in the region, the fact that the four high-D SNPs achieved high-D status in all four of the human populations examined (Figure 1B) suggests that these high-D SNPs appeared before the divergence of the human populations. Coalescence-based dating was carried out for the four high-D SNPs based on two different mutation rates (see Methods). The mutation rate estimated from human-chimpanzee divergence based on a constant molecular clock [25] yielded age estimates for these SNPs (Table S6A) far older than the generally accepted time of human speciation, further testifying to the process of positive selection acting on the region. On the other hand, an accelerated mutation rate equal to that of fast-evolving human mitochondrial DNA [26] yielded age estimates of 139.91±10.09 Kya for rs6556547, 113.31±18.35 Kya for rs187269, 92.82±19.06 Kya for rs1816071, and 69.44±20.14 Kya for rs1816072 in coalescence-based calculations with the Rho index [27] (Table S6; Figure 4). These age estimates, within the time frame between the origin of the anatomically modern human around 150 Kya [28] and migrations out of Africa around 60 Kya [29], gave evidence to a fast rate of evolutionary change, driven by the positive selection, comparable to that of mitochondrial DNA.

### Population variations in allelic and haplotype frequencies

Pronounced population variations were observed with respect to the frequencies of alleles (Figure 1 and Table S1) and haplotypes (Figure 2 and Table S3) among the Yi6AH151 SNPs. The D-allele frequency of the youngest high-D SNP rs1816072 was considerably lower in AF (31.2%) than in the three non-AF populations (57.1–63.9%). This was even more the case with H56, which displayed a frequency of 18.1% for AF compared to 53.4–63.6% for the three non-AF populations. These findings suggest that the accumulation of the D-alleles and HG-D began not long prior to the migrations of the non-AF populations from Africa approximately 60 Kya [30], and continued after the separation of the different populations.

**Figure 5:**
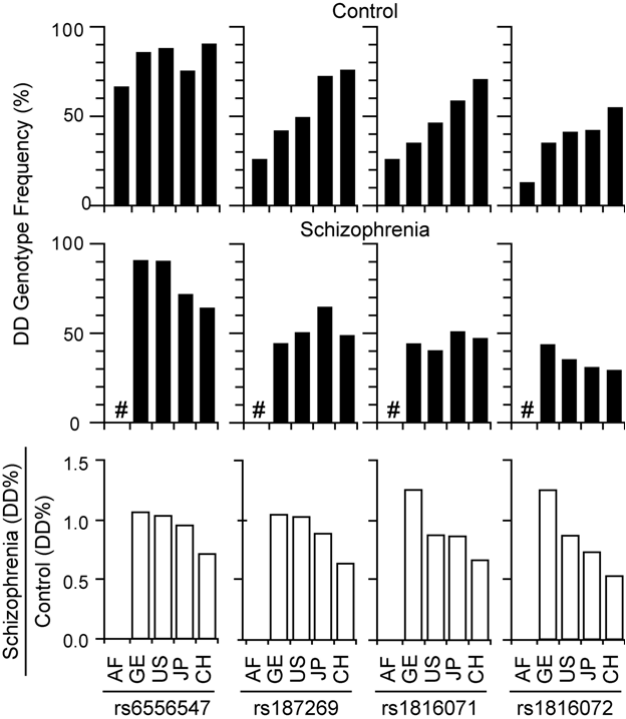
Homozygous D-allele genotype (DD) frequencies for the four high-D SNPs in controls (top) and schizophrenics (bottom) (Table S5). These DD frequencies are plotted for AF, GE, US, JP and *Han* Chinese from Shanghai (CH) with the four high-D SNPs arranged from left to right in a descending order of their estimated ages. To avoid the potential influence from any gender effect, only male samples were employed in this controls-schizophrenics comparison. The absence of schizophrenic samples of AF origin is indicated by “#”.

The evolutionary history of the region surrounding Yi6AH151 could be reconstructed from the male genotype frequencies from the AF and the three non-AF populations together with the Chinese cohort (CH) employed in the initial report on *GABRB2*-schizophrenia association [4] (Table S5). In Figure 5, the homozygous D-allele genotype (DD) frequencies of from left to right the oldest rs6556547, the second oldest rs187269, the third oldest rs1816071 and the youngest rs1816072 in the male control samples from these five populations are plotted in the order of AF-GE-US-JP-CH in accordance with the order of ascending DD frequencies for the three younger SNPs. This ascending order coincided largely with the order of genetic distances of these non-AF populations from AF, which placed the East Asia-Africa distances much greater than the Europe-Africa distances [31]. The similarity of the monotonic ascending DD-frequency gradients for the three younger SNPs is consistent with the identified positive selection being a recent and possibly ongoing process. Comparable DD frequency gradients were not demonstrated for the female samples which were more limited in sample size. The rs6556547 SNP did not exhibit a monotonic ascending DD-frequency gradient, owing at least in part to the high DD frequency of 66.7% (Table S5) even in the AF population, which is keeping with the D-allele of this SNP being the oldest among the four high-D SNPs.

In contrast to the controls, the 436 male schizophrenic samples did not conform to a monotonic ascending DD-genotype frequency gradient for any of the four high-D SNPs (Figure 5 and Table S5). In fact, a modest monotonic descending gradient was displayed by rs1816072, and a flat-top descending gradient by rs6556547. The divergent behavior of the control and schizophrenic DD-frequencies becomes particularly pronounced in the schizophrenia/control plot, which yielded a monotonic descending gradient for all four high-D SNPs. It indicates a relationship between DD-frequencies and schizophrenia that diverges from that between DD-frequencies and the controls, in accord with the association of the SNPs in this genomic region with schizophrenia observed at the genotype level or haplotype level or both [4], [9].

### ATP-dependences of receptors containing the two β_2_-isoforms

Functionally, it has been demonstrated in a separate Caucasian cohort that the presence of the derived alleles of the three younger high-D SNPs rs187269, rs1816071 and rs1816072 in either homozygote or heterozygote forms significantly increased both the total β_2_ mRNA expression of the combined alternatively-spliced long and short β_2_ isoforms, and the long/short β_2_ isoform ratio, relative to the homozygote forms of the ancestral alleles (the fourth high-D SNP rs6556547 was not included in the analysis) [12]. An increase in total β_2_ subunit expression would enhance GABA_A_ receptor functions and therefore the level of inhibitory transmissions in the CNS, the majority of which are mediated by β_2_-containing GABA_A_ receptors. On the other hand, an increase in the long/short β_2_ isoform ratio would bring about a greater run-down of GABA_A_-mediated electrophysiological current upon repetitive GABA stimulation in the absence of externally added ATP [12].

**Figure 6:**
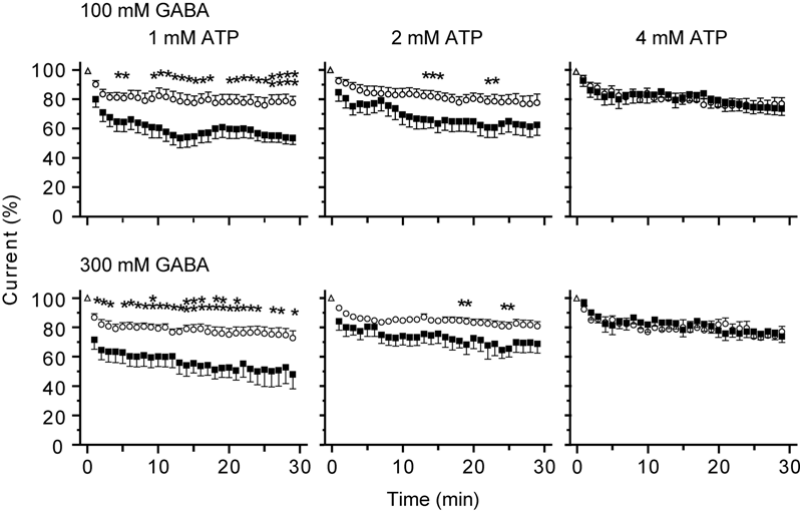
Variations of GABA-potentiated current rundowns with infused ATP. Currents were measured upon 30 successive additions of GABA at 1-minute intervals as described in Methods. Electrophysiological currents, expressed as a percentage of the peak current (▵) measured upon initial response to stimulation by 100 µM (top row) or 300 µM (bottom row) GABA, were measured for α_1_β_2L_γ_2L_ (▪) and α_1_β_2S_γ_2L_ (○) receptors with intracellular infusion of 1 mM, 2 mM or 4 mM ATP. Each data point represents average±standard error recorded from 7–10 cells from at least six independent transfections. Responses that were significantly different between the α_1_β_2L_γ_2L_ and α_1_β_2S_γ_2L_ receptors based on the two-tailed Student's *t*-test are marked by asterisks (*, *P*<0.05; **, *P*<0.01).

The pivotal role of energy status in the current run-down induced by repetitive GABA stimulation was confirmed in the present study by the results in Figure 6, where raising the intracellularly infused ATP from 1 mM to 4 mM was found to reduce the amplitude of the run-down. Since this reduction was much greater with the long β_2_ isoform (β_2L_)-containing receptors compared to the short β_2_ isoform (β_2S_)-containing receptors, the difference in current run-down between the two kinds of receptors became narrowed at 2 mM compared to 1 mM ATP, and practically eliminated at 4 mM ATP. Thus the long/short β_2_ isoform ratio was confirmed as an important determinant in the energy regulation of GABA_A_ receptor function. Such energy regulation of GABA_A_ receptor function could contribute for example to the well-known heightening of alertness by hunger in contrast to the relative lethargy induced by satiety: hunger cuts down ATP supply, thereby inducing GABA-current rundown and decreasing inhibitory CNS neurotransmissions mediated by long β_2_ isoform-containing GABA_A_ receptors. It follows that enhancement of the long/short β_2_ isoform ratio by the derived alleles of the high-D SNPs would reduce such inhibitory CNS neurotransmissions under normal conditions, but much less so under conditions of energy deprivation.

## Discussion

Within the schizophrenia-associated 3,551-bp region of human *GABRB2*, the human lineage displayed accelerated evolutionary adaptation. The high DD-frequencies of the oldest high-D SNP rs6556547 in the control samples, ranging between 70.5–87.1% for the five human populations (Table S1 and Table 1 in [4]) signaled strong positive selection of the derived allele of this SNP over the ancestral allele found in the chimpanzee. For the three younger high-D SNPs rs187269, rs1816071 and rs1816072, the DD-frequencies were not as high, but the positive selections of derived over ancestral alleles were just as evident. These positive selections are striking in view of the low sequence divergence among primates in this region. They were shown to be statistically significant upon testing by stringent criteria (Table S4), and found confirmation in the lower diversity of the haplotypes containing the D-allele of rs1816072 (Figure 2, Figure 3 and Table S2). They might be facilitated by the human-specific Alu Yi6AH151 insertion in the region; insertions of this type are known to increase homologous recombinations and mutations, on which selection could act [32], [33]. The dissimilar enrichments of the derived alleles displayed by the different populations suggest that the positive selections of these alleles, especially the younger ones, are recent and likely ongoing. Based on the age estimates of the four derived alleles obtained using the mitochondrial DNA mutation rate as a proximate, the time-span of these selection processes overlapped with the time frame of Mellars' proposed history of modern human evolution [29], with the emergence of the four high-D SNPs broadly coinciding with human population expansion in, and dispersal from, Africa (Figure 4).

Since the four high-D SNPs occur in the intronic regions of *GABRB2*, the impact of their allelic status may be expected to be regulatory in nature. In keeping with this expectation, their derived alleles were found to increase total β_2_-expression and to enhance inhibition of GABA_A_ function upon ATP depletion [[12]; Figure 6]. These two opposing effects of the derived alleles could be fundamental to the differences between populations. Because β_2_-containing GABA_A_ receptors mediate the majority of CNS inhibitory neurotransmissions, the derived alleles would increase the overall activities of CNS inhibition by increasing total β_2_-expression. At the same time, however, they would also accentuate the inhibition of GABA_A_ function under conditions of energy deprivation as occasioned by food shortages. Accordingly the strength of the positive derived-allele selections in any population would depend on the level of GABA_A_-mediated CNS inhibitions required by the population, as well as the historical pattern of famines and severe food shortages in the course of its evolution. Various environmental, social and other population-specific factors could affect the required level of GABA_A_-mediated CNS inhibitions. In baboons, negative modulation of GABA_A_ receptor produced effects that varied with the social status of the subject [34]. Among humans as well, evidence suggests that social organization could influence behavior pertinent to survival and evolution [35], [36]. The distance of the migrations out-of-Africa, the size of the populations participating in these migrations, and impact of agriculture [37] are other determinants that could favor the evolution of dissimilar GABA_A_ activity profiles in different populations. Consequently, the evolved frequencies of the derived alleles of the high-D SNPs can not be expected to be the same for different populations.

The schizophrenia-relevance of the positive selection of the derived genotypes is suggested by the drastic differences between schizophrenia and control in their DD-frequency profiles (Figure 5). The control samples showed an indefinite trend for the oldest high-D SNP, but an upward gradient for the three younger high-D SNPs, in the DD-frequency plots over the AF, GE, US, JP and CH populations, providing a useful evolutionary snapshot of the selection process. In contrast, an indefinite trend (rs187269 and rs1816071) or downward trend (rs6556547 and rs1816072) was observed over these five populations for the schizophrenic samples; the reasons for the two downward gradients yet remain to be understood, but it is not ruled out that assortative mating, which has been observed in schizophrenia [38], might be a contributing factor. The schizophrenia/control ratio in DD-frequency thus decreased monotonically for all four high-D SNPs over these five populations, clearly establishing that the positive selection manifested by the controls was substantially weakened among the schizophrenics. Although the ratio exceeded unity in the GE samples, was close to unity in the US samples, but less than unity in the JP and CH samples, the schizophrenic/control ratios for all four SNPs conformed to a similar descending gradient over the GE-US-JP-CH populations. This uniformity shown by the four high-D SNPs suggests that the nature of the difference between control and schizophrenic samples was basically comparable for the four individual high-D SNPs.

An *inhibition demand* hypothesis may be proposed to address the biological basis of the overall positive selection of derived genotypes in *GABRB2*, and its potential significance to the etiology of schizophrenia and possibly also other psychiatric disorders involving GABA_A_. The proposal consists of: (a) a balance between CNS excitations and inhibitions is essential to mental health; (b) since there is only one inhibitory neurotransmitter, GABA, in contrast to several excitatory neurotransmitters in the brain, the GABAergic system is among the systems that are stringently subjected to evolutionary adjustment; (c) in general, human genetic evolution is slow relative to a rapidly changing environment that imposes increasing demand on CNS inhibitory functions; (d) the derived genotypes, which influence the regulation of the GABAergic system, tend to be reduced in schizophrenics; and (e) such and other alterations affecting the GABAergic system could contribute to schizophrenia etiology.

While purifying selections are often encountered eliminating the rare, deleterious mutations responsible for mendelian diseases, positive selections of protective derived alleles are increasingly observed in common disorders. For example, there are indications of positive derived-allele selection in the *AGT* gene for angiotensinogen [39] and *CYP3A5* gene for cytochrome P450 [40] protecting against hypertension, and similarly positive derived-allele selection in the *CAPN10* gene for calpain 10 [41] and *PPARgamma* gene [42]–[44] protecting against type-II diabetes. These positive selections, consistent with the *sodium-retention* hypothesis for hypertension [45] and *thrifty-gene* hypothesis for diabetes [46], respectively, may be interpreted in terms of an ancestral-susceptibility model for common diseases [44] which proposes that, on account of shifts of environment and lifestyle from ancient to modern times, originally harmless or even beneficial ancestral alleles could enhance disease susceptibility in the modern populations, such that protective derived alleles providing improved adaptation to the altered circumstances are positively selected. In this regard, it is noteworthy that because common diseases are usually multigenic in nature, the divergence between modern and ancestral circumstances that is beneficially addressed by the protective derived alleles of a disease-susceptibility gene could arise not only from shifts of environment and lifestyle. It could also arise within the body from alterations in the allelic status of other interacting, co-evolving genes, causing the ancestral alleles to interact suboptimally with other genes in the modern population. For common diseases not expressly related to diet or lifestyle, such internal alterations in the genomic context of a susceptibility gene could be as important as, or even more important than, external shifts of environment and lifestyle.

In conclusion, because of changes between ancient and modern populations with respect to the genomic context in which *GABRB2* is embedded, and with respect to environment and lifestyle, the ancestral alleles of the high-D SNPs rs6556547, rs187269, rs1816071 and rs1816072 in *GABRB2* have become functionally inadequate in the modern populations. This inadequacy has brought about recent and possibly ongoing positive selections for their cognate derived alleles. Because of the dissimilar evolutionary paths undergone by different human populations, each population has acquired through evolution its own optimized capacity of GABAergic receptors and response of these receptors to energy regulation, as determined by among other factors the derived alleles of the high-D SNPs. The occurrence of positive selection, observed for the first time for any schizophrenia susceptibility genes, in *GABRB2* points to a fundamental role of GABA_A_ function in schizophrenia etiology, as well as the potential usefulness of searching for positive selection among various susceptibility genes for schizophrenia and other complex disorders.

## Methods

### Human subjects

A total of 1,179 individuals (Female 345; Male 834), including 743 unrelated non-schizophrenia subjects (Female 345; Male 398) and 436 schizophrenic male patients but not including relatives in trios, from four different ethnic populations, African (AF), German Caucasians (GE), Caucasians of USA (US) and Japanese (JP) were studied.

For the JP and GE samples, details of sample source and diagnostic procedure were described in Lo *et al*. [9]. In brief, the JP samples consisted of 207 unrelated control subjects (Female 105; Male 102) and 210 unrelated schizophrenia male patients. The GE samples consisted of 190 unrelated control subjects (Female 76; Male 114) and 119 unrelated male schizophrenics of systematic subtype. All schizophrenia patients were in-patients and fulfilled the diagnostic criteria for schizophrenia according to the fourth edition of Diagnostic and Statistical Manual of Mental Disorders [47]. The GE patients belonged to the systematic schizophrenia subtype, the most severe form of schizophrenia, on the basis of Leonhard's classification of endogenous psychoses [48]. Written informed consent was obtained from each subject. Approval for the study was obtained from the ethnical committees of Kurume University for the JP samples and of University of Würzburg for the GE samples.

A total of 370 US samples consisted of 263 unrelated control subjects (Female 127; Male 136) and 107 schizophrenia male patients. Recruitment of the samples was as described [49]. Briefly, the patients were evaluated using the semi-structured diagnostic interview scale called the Diagnostic Interview for Genetic Studies [50]. This information was combined with medical records and available information from relatives. Consensus diagnoses were made using DMS-IV criteria. The controls were drawn unscreened from neonates from a local hospital without any demographic details apart from gender and reported maternal ethnicity. The study was approved by the University of Pittsburgh Institutional Review Board. Written informed consent was obtained from all participants except for the neonate controls in accordance with IRB guidelines.

For the AF population, DNA samples of 25 unrelated individuals and 30 parent-offspring trios were obtained from Coriell Cell Repositories (Camden, NJ). The 25 unrelated individuals (Female 9; Male 16) included eight from North of Sahara (Female 2; Male 6; Panel ID HD12), seven from South of Sahara (Female 2; Male 5; Panel ID: HD11), five Biaka African-Pygmy from Bagandu in the Central African Republic (Female 3, Male 2; Repository Number NA10469–NA10473), and five Mbuti African-Pygmy from Ituri Forest in northeast Zaire (Female 2; Male 3; Repository Number NA10492–NA10496). All members of each of the 30 parent-offspring trios from Yoruba in Ibadan, Nigeria (Female offspring 7; Male offspring 23; Panel ID: HAPMAPPT03) were genotyped and yielded successful genotyping percentage greater than 90% except for two mother samples (Repository Numbers GM19209 and GM19099), which accordingly were not included in present study. The accuracy in phase estimation of haplotypes for parents was confirmed by genotype data from the offspring. Only data of unrelated parents (Mother 28; Father 30) were used in the statistical analysis.

### Genomic DNA of non-human primates

Non-human primate genomic DNA samples obtained from Coriell Cell Repositories (Camden, NJ) included (with Coriell Repository Numbers): *Macaca arctoides* (stumptail macaque, NA03443), *Macaca nemestrina* (pigtailed macaque, NG07921), *Macaca nigra* (celebes crested macaque, NG07101), *Macaca fascicularis* (crab-eating macaque, NA03446) and *Erythrocebus patas* (patas monkey, NG06254).

### Polymerase chain reaction and sequencing

To fully sequence the 3,551 base pairs (bp) genomic region flanked by the two schizophrenia-associated SNPs rs6556547 and rs187269 in *GABRB2* [4], [9], a 7.4-Kb genomic region starting from 2,148 bp upstream of Exon 9 to 519 bp downstream of Exon 10 was generated by polymerase chain reaction (PCR) and served as first PCR template for amplification of two nested-PCR products. List of potential primers for PCR and resequencing were designed using the Primer 3 program [51]. The sequences of primers, designed based on chromosome 5 contig NT_023133.12, are listed in Table S7. Specificity of each potential primer was checked with BLASTN, an alignment tool of the National Center for Biotechnology Information (www.ncbi.nlm.nih.gov/BLAST/). Only specific pairs of primers, with less than 5 hits to the human genome and none of them in GABA_A_ receptor genes other than *GABRB2*, were accepted. Together with the relatively long length (7.4 Kb) of the first PCR product and the employment of nested-PCR, this level of primer specificity reduced the probability of amplifying paralogous sequences in the genome.

PCR amplification of the 7.4-Kb region was performed in a 20 µl mixture containing 70 ng of genomic DNA, 2 µl of 10× PCR buffer for KOD Hot-Start DNA Polymerase, 20 µM of each dNTP, 0.4 mM of MgSO_4_, 0.12 µM of each primer and 0.5 U of KOD Hot-Start DNA Polymerase (Novagen, Madison WI). PCR conditions were optimized using gradient PCR to ensure homogeneity of the amplified products. PCR consisted of initial polymerase activation at 95°C for 3 min, followed by 36 cycles each of 30 sec at 95°C, 30 sec at 62°C and 5 min and 30 sec at 68°C, and a final extension step at 68°C for 5 min and 30 sec. Post-PCR product was immediately purified by ethanol precipitation and recovered in 1× Tris-Cl (USB, Cleveland, Ohio) and ethylene-diamine-tetra-acetic acid (EDTA) (Invitrogen Corporation, Grand Island, NY) buffer, as described in Lo *et al*. [4].

Two nested-PCR fragments, one from 2,148 bp upstream to 498 bp downstream (fragment A), and the other from 342 bp upstream to 2,039 bp downstream (fragment B), of exon 9 were amplified using the 7.4-Kb purified PCR product as template. Each nested PCR reaction contained 0.6 µl of the 7.4-Kb purified PCR product, 75 nM of each primer, 50 nM of each dNTP, 2.5 mM of MgCl_2_ and 1 U of *Taq* DNA polymerase (Amersham Bioscience, Uppsala, Sweden) in a final volume of 20 µl. The nested-PCR reaction consisted of an initial denaturation at 94°C for 2 min, followed by 35 cycles each of 30 sec at 95°C, 30 sec at 58°C, 90 sec at 72°C, plus a final extension step at 72°C for 5 min. After nested PCR, the product in each instance was resolved on 1.2% agarose gel, stained with 0.5 µg/ml of ethidium bromide, and examined under UV to confirm the presence of PCR product of expected size and absence of non-specific products. The stringency of nested-PCR amplification exceeded that for normal PCR, further eliminating non-specific products. The nested-PCR products were purified and recovered as for the first PCR product.

### SNP discovery and genotyping

Both SNP discovery and genotyping were carried out by resequencing the nested-PCR products. For each of the nested PCR Fragment A and B, four sequencing primers were employed (Table S7). Each sequencing reaction contained 3 µl of sequencing buffer, 0.5 µl of BigBye® Terminator version 3.1 (Applied Biosystems Inc., Foster City, California), ∼100 ng purified nested PCR products and 1 mM sequencing primer. Each cycle of sequencing reaction consisted of initial denaturation at 96°C for 1 min, followed by 25 cycles each of 10 s at 96°C, 5 s at 50°C, and 4 min at 60°C. Ethanol precipitation was used to clean-up the post-sequencing products as for the PCR products. Each air-dried sequencing sample was dissolved in 10 µl Hi-Deionized Formamide (Applied Biosystems Inc., Foster City, California), denatured at 95°C for 1 min and immediately held at 4°C prior to sequencing with a Model 3100 Genetic Analyzer (Applied Biosystems Inc., Foster City, California).

Sequence chromatogram alignment-based SNP discovery and genotype calling were carried out using the software package PolyPhred version 4.2 [52]. All genotyping results were manually confirmed by at least two independent researchers. All analyzed SNPs were located within the high-quality region (Quality Value ≥20), and occasional low-quality passes were re-sequenced.

### Inference of haplotype phase

Inference of haplotypes from the genotype data was performed using PHASE version 2.1 as described [53]. The haplotype phases of both parents in AF and US family-trios were estimated based on the genotype data of the corresponding offspring using the P1 option of the program. The inferred haplotypes of AF and US parents were used as phase-known samples in the haplotype phase estimation of population-based genotype data in order to increase the accuracy of haplotype reconstruction. Input of genotype data of unrelated individuals was then placed into the program to infer haplotype phase under the default settings for five independent runs, except that the final iteration was carried out with a 10-fold longer running time. Thus the inferred haplotypes were obtained from five independent runs giving the best average goodness of fit.

### Construction of hypothetical human ancestral sequence

Alignment of human and non-human primate sequences for a ∼1.8-Kb region upstream of Exon 9 of *GABRB2* was performed using ClustalW [54]. This revealed a human-specific Alu insertion in Intron 8 of *GABRB2*. Similarity between sequences was visualized using VISTA [55].

To characterize the sub-family identity of the human-specific Alu insertion in Intron 8 of *GABRB2*, its sequence was searched against the repetitive elements library RepBase using the program RepeatMasker [56]. Consensus sequences of Alu subfamilies and the sequence of the inserted Alu were aligned using ClustalW [54]. To obtain the consensus phylogenetic tree, the SEQBOOT, DNAPARS and CONSENSE programs in the PHYLIP software package [57] were employed. The consensus phylogenetic tree was displayed using TreeView version 1.6.6 [58].

The hypothetical human ancestral sequence of the 3,551-bp region of *GABRB2* was constructed in two stages. For SNPs on the sequences flanking the human-specific Yi6AH151, chimpanzee sequences were used as outgroup to infer their ancestral alleles. For SNPs within Yi6AH151, consensus sequence of the Alu Yi6 subfamily was used for this purpose.

### Summary statistics of mutation parameters

The Hudson, Kreitman and Aguadé's test [18] is based on the neutral molecular evolution expectation that DNA sequence polymorphism within a species and DNA sequence divergence between species, will be proportional to the neutral mutation rate [59]. By comparing the number of nucleotide differences between and within species for the 3,551-bp region against the genome average estimates using DnaSP, the rate of evolution in this region could be tested for departure from neutrality.

DNA diversity level among human sequences was measured using the program DnaSP to yield four mutation parameters: haplotype diversity *H_d_* [60] equivalent to gene heterozygosity, nucleotide diversity π [61] measuring the average number of pairwise differences, Watterson's θ-_W_ [62] based on the number of segregating sites, and θ-_H_ [63] weighting the presence of high-frequency derived variants. Under the standard neutral model of a random-mating population of constant size [59], these genetic mutation parameters would each approach θ  = 4*Ne*µ, where *Ne* is the diploid long-term inbreeding effective population size and µ is the mutation rate per generation.

The four summary statistics Tajima's *D* [64], Fu and Li's *D* and *F* [65] and Fay and Wu's *H* [63] were evaluated regarding the site-frequency spectrum for or against an assumption of neutrality. Departures from the neutral model are usually attributed to selective or demographic effects. The Tajima's *D* test compares θ-_W_ to π. The Fu and Li's *D* and *F* tests compare θ-_W_ and π respectively to the number of derived unique mutations, which represent the mutations on external branches of the tree. The Fay and Wu's *H* test compares π to θ-_H_, and is sensitive for detecting an excess of high-frequency-derived alleles, from a hitchhiking effect under positive directional selection. For the Fu and Li's *D* and *F* and Fay and Wu's *H* tests, an outgroup sequence is required to define the ancestral status of alleles. The hypothetical human ancestral sequence was used as the outgroup sequence.

The significance of any difference in Fay and Wu's *H* value between HG-N and HG-D was tested with 10^5^ permutations. In each permutation, the N and D alleles of SNP rs1816072 were randomly exchanged between the two groups of haplotypes, and absolute differences in Fay and Wu's *H* value were calculated between the two groups. The number of permutations out of the total of 10^5^ permutations yielding a greater difference in value between HG-N and HG-D were counted to give the probability value *P*.

### Coalescence-based neutrality test with demographic models

The simulation method based on the coalescence process was employed using the *mksample* program [66]. For each human population, the observed number of chromosomes and segregating sites were specified to generate 10^5^ simulation datasets to test the significance of Fay and Wu's *H* and Tajima's *D* values and the probability of positive selection of the major haplotype for each of the demographic models. Recombination rate and gene conversion were stringently set at the 10^−8^ per generation close to the genome average, and an average tract length of 100 bp, respectively, to minimize type I error.

The significance of Fay and Wu's *H* and Tajima's *D* values in each population was calculated using simulations of various demographic models under the assumption of neutral evolution. For each population, the number of simulation datasets showing Fay and Wu's *H* or Tajima's *D* values smaller than the observed *D* or *H* values was counted. On the other hand, in order to access the statistical probability of a history of positive selection at the major haplotype H56 in human populations, a null hypothesis of observing a haplotype containing four derived alleles having the same or a higher frequency than the observed frequency of H56 in neutral evolution with various demographic histories was tested.

A total of thirty-three demographic models, namely M1 to M33 were examined. The models of M1 to M9 embodied the effects of population growth and substructure as described by Evans *et al*. [21]. M10 to M25 were models with population growth and substructure based on Currat *et al*. [23]. M25 to M29 were population bottleneck models based on the demographic analysis of multiloci data by Voight *et al.* [22]. M30 to M33 were the low-migration models as described by Garrigan and Hammers [24].

The parameters for Evans' models [21] M1 to M9 were as follows: **M1**, a constant effective population size of 10^4^; **M2**, exponential population expansion since 5,000 generations ago from an initial population size of 10^4^ to a present population size of 10^7^; **M3**, exponential population expansion since 1,000 generations ago from an initial population size of 10^4^ to a present population size of 10^7^; **M4**, a severe bottleneck starting 5,000 generations ago reduced the population size instantly from 10^4^ to 10^3^, which remained constant until 2,500 generations ago, when the population expanded exponentially to a present size of 10^7^; **M5**, five consecutive bottleneck events started at 7,000 generations ago, in each instance reducing the population size from 10^4^ instantly to 10^3^, following by constant size for 500 generations and subsequent recovery by exponential growth back to 10^4^ in the ensuing 500 generations; starting at the end of the fifth bottleneck at 2,500 generations ago, the population expanded exponentially to a present size of 10^7^; **M6 to M9**, having a population structure where the total number of chromosomes for each human population was split equally into two to five subpopulations, with 1 migration per generation between any two populations under constant population size condition.

In Currat's models [23], a subpopulation split off from a core population at 1,000 generations before the present, and grew exponentially from an initial effective population size (*n*_s_) to the present size of 10^7^ individuals. For each model, a range of *n*_s_ from 10 to 10^4^ was tested. For the stable core models **M10 to M13** among them, the effective population size of the core population remained constant at 10^5^; bottleneck severities ranged from 0.0001 for *n_s_* = 10 to 0.1 for *n_s_* = 10^4^. For the growing core models **M14 to M17**, the population core grew from an initial size of 10^4^ individuals at 1,000 generations ago to 10^7^ at present. For the extended growing core models **M18 to M21**, the core population grew from 10^4^ individuals at 5,000 generations ago to the present 10^7^. For the early fission models **M22 to M25**, a subpopulation split from a core population of *n*_s_ at 5,000 generations ago and remained constant in size until 1,000 generations ago, and grew thereafter to the present 10^7^, while the core population grew from 10^4^ individuals at 5,000 generations ago to the present 10^7^.

**M26** to **M29** were population-bottleneck models based on Voight *et al*. [22]. For all of these four models, a bottleneck event occurred at 1,600 generations ago (t_start_), when an ancestral population (N_A_) of 10^4^ was reduced to a size of 10^3^ for M26 (bottleneck severity, *b* = 0.1), 4×10^3 ^for M27 (*b* = 0.4), and 50 for both M28 and M29 (*b* = 0.005). After a period of t_dur_, the population grew exponentially back to its original size of 10^4^. The t_dur_ was 400 generations for M26, zero for M27, 600 for M28, and 300 for M29.

The low-migration models **M30 to M33**, based on Garrigan and Hammers [24], described a structured ancestral population of 2,000 chromosomes, which admixed with two other demes of the same size at a low-migration rate (4Nm = 0.5) 32,000 generations ago (800 Kya); a bottleneck event occurred at 1,600 generations ago as described in M26 to M29, except that the population size recovered to 20,000 chromosomes.

### Estimation of SNP coalescence age

Two established mutation-based methods were employed to estimate the coalescence age of SNP: a) using the molecular clock derived from the divergence between human and chimpanzee to determine the time to the Most Recent Common Ancestor MRCA [25]; and b) using the Rho (ρ) index to define the age in unit of mutation from MRCA to the descendent haplotypes of SNPs [27], and the mutation rate of human mitochondria DNA (mtDNA) [26] to obtain the estimated coalescence age of SNPs. Both approaches were suggested to be unbiased by demographic history [25], [27].

In the first method, the SNP age was estimated by the mutation-based method of Thomson *et al.* [25]. Average number of mutations per base in lineages from the MRCA to haplotypes carrying the D allele of SNP (*d*) for each human population was calculated using the DnaSP version 4.10 [67]. By comparing human and chimpanzee sequences in this region, the human-chimpanzee nucleotide divergence (*D*) in the region was estimated for human populations in mutations per base. Assuming a human-chimpanzee divergence time (*T*_split_) of 6 Mya, the estimated mutation rate per site per year (μ) was obtained by the formula μ = *D*/(2*T_split_). The time to MRCA (TMRCA) in years was obtained by the formula TMRCA = d*μ.

In the second method, the age of haplotypes carrying derived alleles of SNPs in terms of the number of mutations was measured by the Rho (ρ) index [27] by means of the program NETWORK version 4.2 (NETWORK website). A phylogenetic tree of inferred haplotypes was constructed using the median-joining method [68]. By defining a root node, i.e. the hypothetical human ancestral haplotype, ρ between the root node and a descendent haplotype or a group of descendent haplotypes, i.e. a haplotype group, was calculated. The ρ statistic represents the average number of mutational changes between the root node and an individual haplotype within the phylogeny relating the intra-allelic diversity of the root node and the haplotypes of interest. Relationship between ρ and TMRCA (*t*) is given by the equation, ρ = μ*t* where μ is the mutation rate per site per year. To estimate the TMRCA, the mutation rate μ for human mtDNA, which is equal to 1 mutation per 20,180 years [26], was employed for converting ρ to *t*. The age of a descendent haplotype is the same as the minimum age estimate for the preceding mutation.

### Expression and electrophysiology of recombinant GABA_A_ receptors

Human embryonic kidney (HEK293) cells were transiently co-transfected with pcDNA3.1-α_1_, pcDNA3.1-γ_2L_ plus either pcDNA3.1-β_2S_ or pcDNA3.1-β_2L_, using the Genejuice transfection reagent (Novagen) as described [12]. Whole-cell patch clamp recordings were carried out on the cells using an EPC9 amplifier (HEKA, Germany) at 36 to 60 hours after transfection. Cells were voltage-clamped at −60 mV. Pipette-to-bath resistance was 3.0–6.0 MΩ when filled with the internal solution containing 140 mM of CsCl, 1 mM of MgCl_2_, 11 mM of EGTA, 10 mM of HEPES, and 1, 2 or 4 mM of Mg^++^-ATP, adjusted to pH 7.3 with CsOH. The external superfusion solution contained 150 mM of NaCl, 3 mM of KCl, 1 mM of MgCl_2_, 1 mM of CaCl_2_ and 10 mM of HEPES, adjusted to pH 7.2 with NaOH [69]. The cells were constantly perfused with the external solution at 5 ml/min.

Repetitive applications of GABA (100 or 300 µM, 5-s duration, 60-s interval) to induce current rundown was initiated when the cells were found to display consistent responses to low concentration of GABA (10 µM, 3-min interval) [70]. Thirty consecutive whole-cell responses to the repetitive applications were evaluated in terms of the observed peak current as percentile initial peak current. The results obtained from 7–10 cells collected from 6 independent transfections were presented in mean±standard error. Two-tailed Student's *t*-test was employed for calculation of statistical differences between receptors containing β_2L_ and those containing β_2S_.

## Supporting Information

Table S1 All twenty-nine SNPs identified from unrelated non-schizophrenic individuals of four different populations.
(0.03 MB XLS)

Table S2 Summary statistics of mutation frequency spectrum for haplotype-groups of rs1816072 in different populations.
(0.02 MB XLS)

Table S3 Compositions and population frequencies of 75 inferred haplotypes.
(0.06 MB XLS)

Table S4 Coalescence-based neutrality test of various demographic models.
(0.04 MB XLS)

Table S5 Population frequencies and frequency ratio of the four high-D SNPs in male controls and schizophrenics.
(0.02 MB XLS)

Table S6 Estimated coalescence age of the four high-D SNPs by mutation-based methods.
(0.03 MB XLS)

Table S7 Primers for PCR and sequencing of the 3,551-bp *GABRB2* fragment.
(0.02 MB XLS)

Figure S1 Sequence similarity between human and 7 non-human primates displayed by VISTA [55]. The sequences of chimpanzee and rhesus were obtained from the NCBI database. The DNA of the five other non-human primates were sequenced over this 1.8 Kb region in *GABRB2*.
(0.82 MB TIF)

Figure S2 An unrooted phylogenetic tree of Alu sequences. (A) The tree was generated from an alignment of the consensus sequences of Alu sub-families and the Yi6AH151 using DNAPARS in the PHYLIP software package [57]. The consensus tree generated from 1,000 replications is labeled with bootstrap values at the nodes and displayed with TreeView [58]. The clade containing human-specific Alu Yi6AH151 and its closest neighbor Alu Yi6 is highlighted in grey. (B) Sequence alignment of Alu Yi6 consensus sequence and Yi6AH151. Refer to Salem et al. [14] for the sequences of all 150 members of Yi6 subfamily.
(0.98 MB TIF)

Figure S3 Plot of estimated recombination rate and location of recombination hotspots in *GABRB2*. The plot is adapted from Genome Browser (http://genome.ucsc.edu/cgi-bin/hgGateway) report, which employed the HapMap Phase II data. The red box indicates the region of *GABRB2* studied.
(0.75 MB TIF)

Figure S4 Summary statistics for all HG-N and HG-D haplotypes. Tajima's *D* and Fu and Li's *D* values are plotted for AF, GE, US and JP.
(0.97 MB TIF)
