# Supplementary figures and images for: Positive Selection within the Schizophrenia-Associated GABAA Receptor β2 Gene
Source: PLoS One. 2007 May 23;2(5):e462. doi: 10.1371/journal.pone.0000462 (PMC1866178; doi:10.1371/journal.pone.0000462)

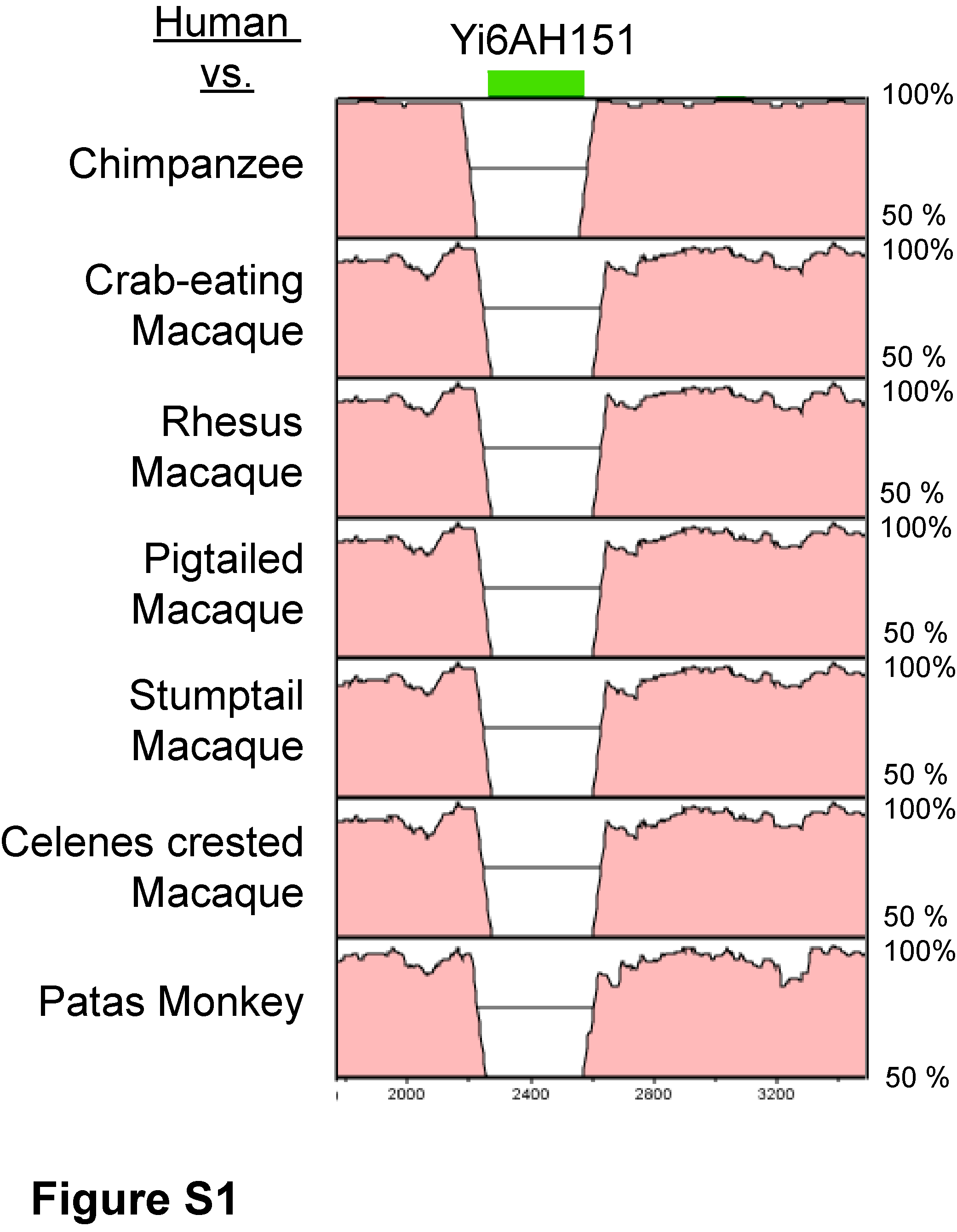

Supplement: Figure S1 — Sequence similarity between human and 7 non-human primates displayed by VISTA [55]. The sequences of chimpanzee and rhesus were obtained from the NCBI database. The DNA of the five other non-human primates were sequenced over this 1.8 Kb region in GABRB2. (0.82 MB TIF) [file pone.0000462.s008.tif]

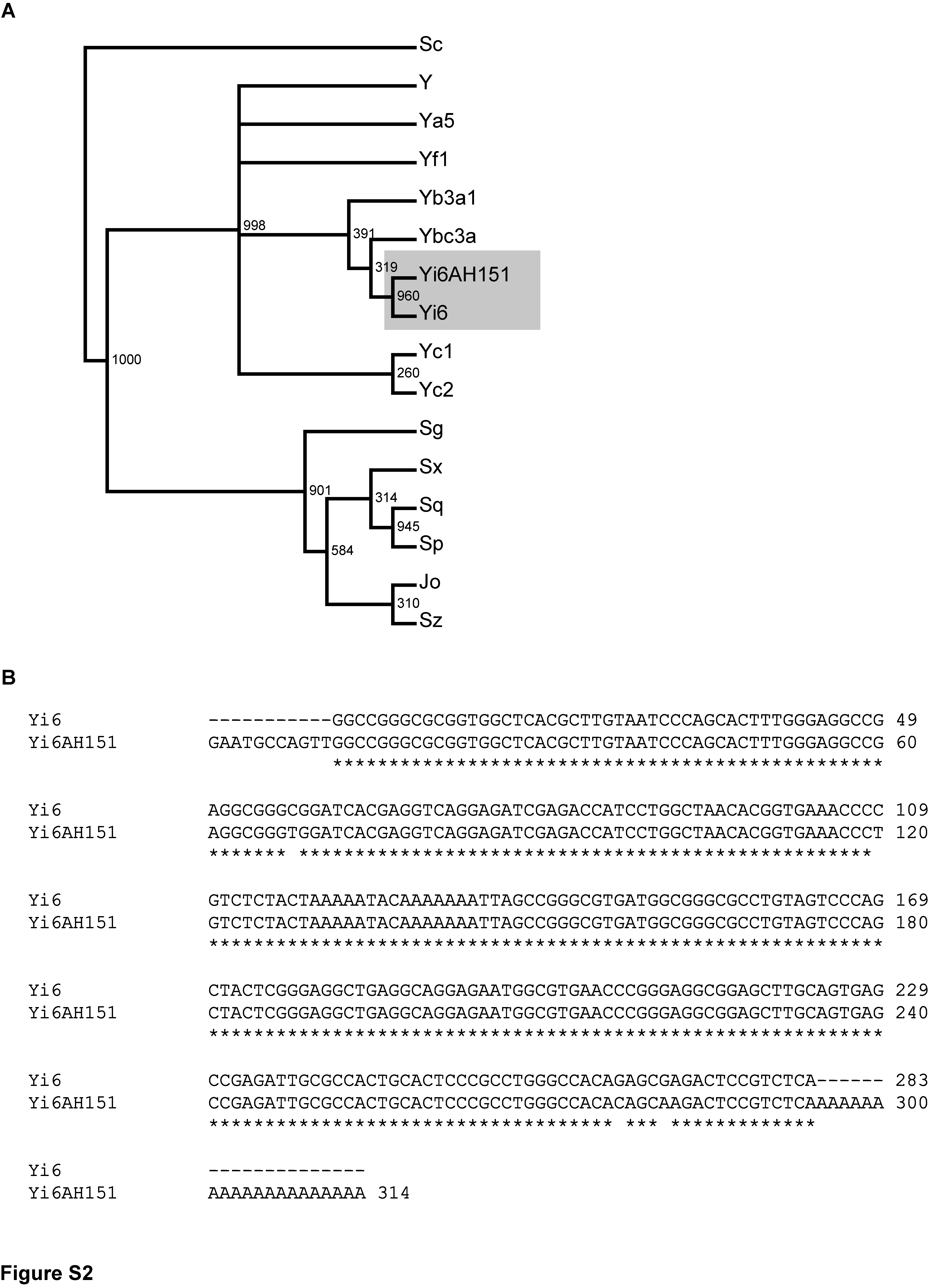

Supplement: Figure S2 — An unrooted phylogenetic tree of Alu sequences. (A) The tree was generated from an alignment of the consensus sequences of Alu sub-families and the Yi6AH151 using DNAPARS in the PHYLIP software package [57]. The consensus tree generated from 1,000 replications is labeled with bootstrap values at the nodes and displayed with TreeView [58]. The clade containing human-specific Alu Yi6AH151 and its closest neighbor Alu Yi6 is highlighted in grey. (B) Sequence alignment of Alu Yi6 consensus sequence and Yi6AH151. Refer to Salem et al. [14] for the sequences of all 150 members of Yi6 subfamily. (0.98 MB TIF) [file pone.0000462.s009.tif]

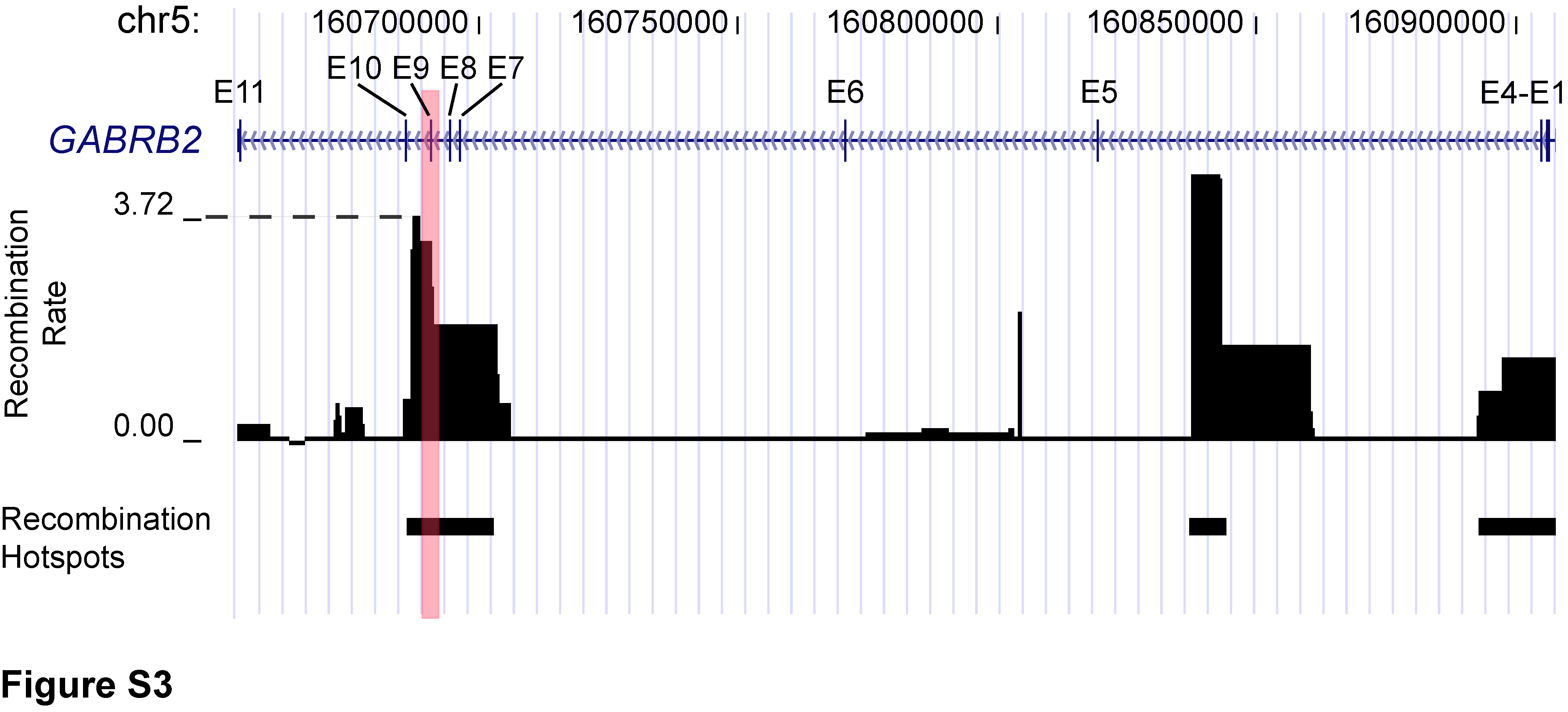

Supplement: Figure S3 — Plot of estimated recombination rate and location of recombination hotspots in GABRB2. The plot is adapted from Genome Browser (http://genome.ucsc.edu/cgi-bin/hgGateway) report, which employed the HapMap Phase II data. The red box indicates the region of GABRB2 studied. (0.75 MB TIF) [file pone.0000462.s010.tif]

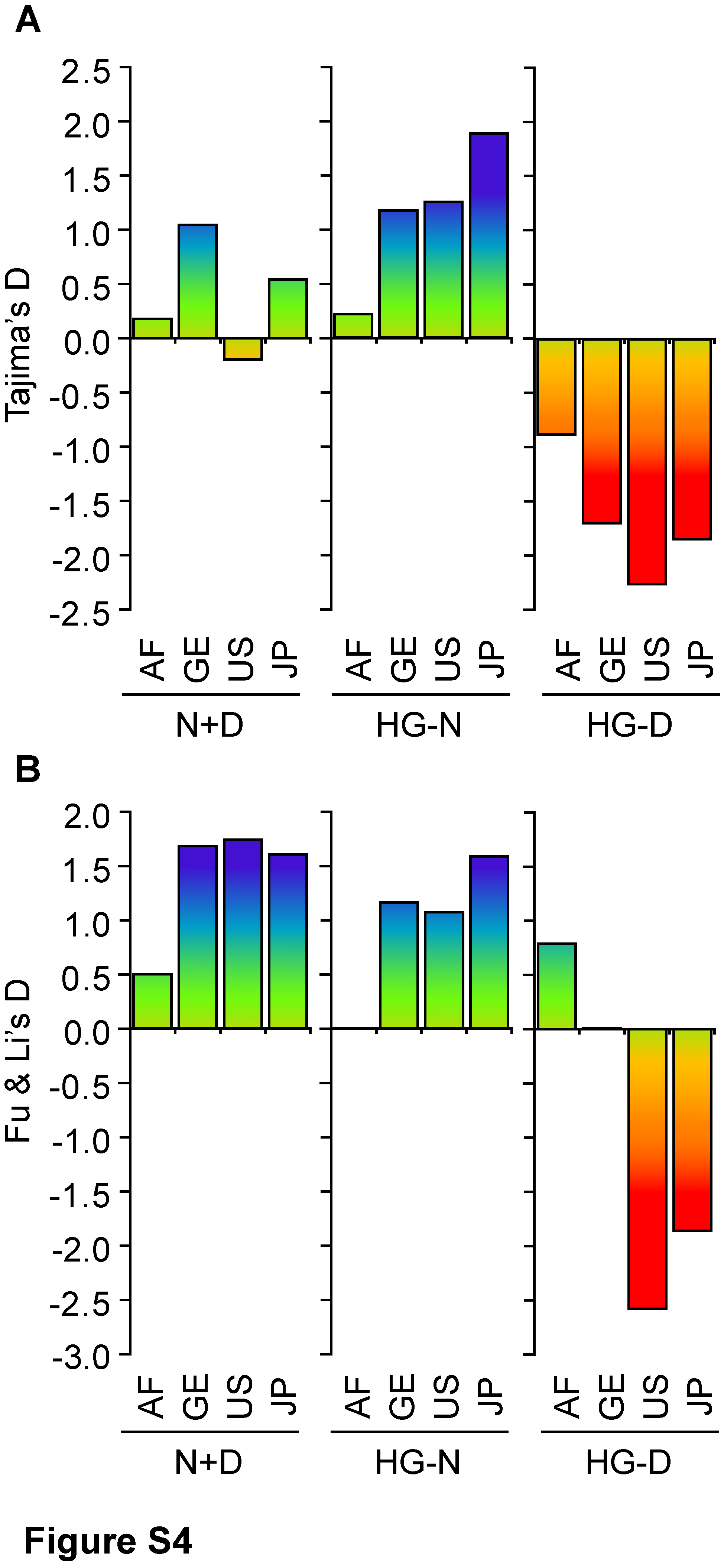

Supplement: Figure S4 — Summary statistics for all HG-N and HG-D haplotypes. Tajima's D and Fu and Li's D values are plotted for AF, GE, US and JP. (0.97 MB TIF) [file pone.0000462.s011.tif]
